# A branched peptide targets virus and host to block influenza virus and rhinovirus entry

**DOI:** 10.1128/aac.00024-25

**Published:** 2025-06-25

**Authors:** Xinjie Meng, Chuyuan Zhang, Xiankun Wang, Jilong Shi, Zixian Song, Purui Ke, Yao Chen, Ruiqing Sun, Yee-Man Lau, Kwong-Man Ng, Chun-Ka Wong, Hung-Fat Tse, Linlei Chen, Kwok Hung Chan, Cyril Chik-Yan Yip, Jie Zhou, Youhua Xie, Shibo Jiang, Kelvin Kai-Wang To, Kwok-Yung Yuen, Hanjun Zhao

**Affiliations:** 1 Shanghai Institute of Infectious Disease and Biosecurity, Shanghai Medical College, Fudan University 12478, Shanghai, China; 2 Centre for Virology, Vaccinology and Therapeutics, Hong Kong Science and Technology Park 567841 , Hong Kong, China; 3 State Key Laboratory for Emerging Infectious Diseases, Department of Microbiology, School of Clinical Medicine, Li Ka Shing Faculty of Medicine, The University of Hong Kong, Hong Kong, China; 4 Cardiology Division, Department of Medicine, The University of Hong Kong, Hong Kong, China; 5 Pandemic Research Alliance Unit at The University of Hong Kong, The University of Hong Kong, Hong Kong, China; 6 Carol Yu Centre for Infection, Li Ka Shing Faculty of Medicine, The University of Hong Kong, Hong Kong, China; IrsiCaixa Institut de Recerca de la Sida, Barcelona, Spain

**Keywords:** antiviral peptide, human defensin peptide, influenza virus, rhinovirus, viral entry

## Abstract

The global burden of influenza virus and rhinovirus, along with significant mortality and severe case reports, underscores the urgent need for new antivirals. Human defensins serve as the first line of defense against viruses; however, the antiviral activity of defensin peptides is often sensitive to salt, which affects their effectiveness. This study investigates a branched human-defensin peptide H30 (4H30) that can more effectively inhibit influenza virus and rhinovirus compared to the linear form of H30. Mechanistic studies reveal that 4H30 binds to influenza HA to aggregate the virus, thereby blocking viral entry. 4H30 can also cross-link H1N1 virus with cell surface glycosaminoglycans to prevent viral release. The dual-functional peptide 4H30 protects mice from the lethal challenge of the A(H1N1)pdm09 virus, demonstrating a high barrier to viral resistance after 15 viral-culture passages in the presence of 4H30. Notably, 4H30 interferes with the low-density lipoprotein receptor (LDLR) to impede the entry of minor group rhinovirus and significantly inhibits rhinovirus replication in RD cells, nasal organoids, and stem cell-derived cardiomyocytes. These findings suggest that the branched peptide 4H30, targeting both the virus and host, can more effectively inhibit influenza and rhinovirus than the linear H30, providing a new avenue for antiviral peptide development.

## INTRODUCTION

Influenza virus can cause seasonal influenza with millions of infections yearly and has caused four pandemics in the past centuries (1, 2). On the other hand, rhinovirus, despite being underestimated in previous years, is now recognized as a significant player in respiratory infectious diseases, and co-infections of influenza and rhinovirus are common in patients (3, 4). Studies suggest that rhinovirus may be responsible for over 50% of upper respiratory tract infections (5). In particular, rhinovirus is the primary virus isolated from hospitalized infants and young children (5, 6). While oseltamivir and zanamivir are FDA-approved drugs against the influenza virus, drug-resistant viruses can emerge through natural evolution or drug treatments (7, 8). Moreover, the suboptimal effectiveness of available anti-influenza drugs against the high pathogenic A(H5N1) and (H7N9) viruses with high mortality rates (> 30%) (9, 10) raised the challenges of the current antivirals. To date, no clinically effective drug has been approved for treating rhinovirus infections. Thus, developing new broad-spectrum antivirals, particularly those administered orally or via inhalation (11), is critical for combating respiratory influenza and rhinovirus infections, especially for the practical administration in outpatients.

Human beta-defensins are widely expressed in multiple organs, especially in respiratory epithelial cells (12). They are consistently expressed in different organs and are induced by microbial infections or suppressed in cancer diseases (13). Defensins have potential as antimicrobial drugs or diagnostic markers (14). However, challenges exist in developing recombinant defensins for drug purposes due to their salt sensitivity (15) and the difficulty of large-scale production by eukaryotic cells. Therefore, nonrecombinant antiviral defensins that are salt-insensitive could be promising candidates for drug development. Human beta-defensin 2 (HBD2) has demonstrated antiviral activity against influenza, respiratory syncytial virus, and other respiratory viruses (16). Nevertheless, the *in vitro* antiviral efficacy of human defensins against SARS-CoV-2 was limited, with few studies demonstrating *in vivo* antiviral activity (17). Branched peptides, known for their high stability *in vivo* (18) and salt insensitivity (19), could potentially enhance the activity of defensin peptides.

In this study, we investigated the antiviral mechanisms and activities of branched-peptide 4H30 (derived from human beta-defensin 2) against influenza virus and rhinovirus to validate its broad-spectrum antiviral activities against different viral families. Our findings revealed that 4H30 aggregated influenza virus and targeted the cellular receptor LDLR of rhinovirus, effectively blocking the entry of both viruses. Moreover, 4H30 effectively blocked influenza virus release by cross-linking H1N1 virus with cell surface glycosaminoglycans and exhibited a high barrier to viral resistance after 15 passages. Subsequent studies showed that 4H30 significantly inhibited A(H1N1)pdm09 replication in mice, protected mice from the lethal challenges of A(H1N1)pdm09, and inhibited rhinovirus replication in nasal organoids and cardiomyocytes. These results suggested that branched 4H30, as opposed to linear H30, could significantly inhibit influenza virus and rhinovirus replication in high salt conditions, highlighting the advantages of 4H30 with potent antiviral activity *in vivo* and broad-spectrum antiviral activity against various respiratory viruses across different families by blocking viral entry.

## MATERIALS AND METHODS

### Cells and viral culture

Madin Darby canine kidney (MDCK, CCL-34) and RD (CCL136) cells obtained from ATCC (Manassas, VA, USA) were cultured in minimal essential medium (MEM) supplemented with 10% fetal bovine serum (FBS), 100 IU mL^−1^ penicillin, and 100 µg mL^−1^ streptomycin. The virus strains used in this study included enveloped influenza A/Hong Kong/415742/2009, A/Hong Kong/415742Md/2009 (H1N1) and H3N2 virus (20), and non-enveloped rhinovirus A1 and rhinovirus A16 (21). For *in vitro* experiments, viruses were cultured in MDCK and RD cells, and for animal experiments, the H1N1 virus was cultured in eggs. Cardiomyocytes are generated from human-induced pluripotent stem cells (iPSCs) as we had previously published (22, 23).

### Peptide synthesis

Human beta-defensin peptide H30 (GAICHPVFCPRRYKQIGTCGLPGTKCCKKP) and 4-branched peptide 4H30 (H30 cross-linked by Lysine at the C terminal) and P9RS (24) were synthesized by ChinaPeptide (Shanghai, China). All peptides were dissolved in water. The solubility of peptides in water is greater than 5 mg mL^−1^. The purity of all peptides was >80%. The purity and mass of each peptide were verified by HPLC and mass spectrometry.

### Plaque reduction assay

The antiviral activity of peptides was measured by plaque reduction assay. Briefly, peptides were dissolved in PBS or 20% PBS. Peptides (0.2-25.0 µg mL^−1^) were premixed with H1N1 virus at room temperature. After 30 min of incubation, the peptide-virus mixture was transferred to MDCK cells for infection at 37°C. At 1 hpi, infectious media were removed, and then 1% low melting agar was added to cells. Cells were fixed using 4% formalin at 2–3 dpi. Crystal blue (0.1%) was added to cells for cell staining to count viral plaques.

### Anti-rhinovirus assay

Rhinovirus was treated with peptide in PBS or 20% PBS, and peptide-treated virus was added to RD cells for infection at 33°C for 1 h. After removing the infectious media, MEM with or without peptide was added to cells for viral culture. Cell lysates or supernatants were collected at 16 hpi or 30 hpi to measure viral RNA using one-step RT-qPCR.

### RT-qPCR assay

Viral RNA was extracted by the Viral RNA Mini Kit (QIAGEN, Cat^#^ 52906, USA). RT-qPCR was performed as described previously (20). Extracted viral RNA was reverse-transcribed to cDNA using PrimeScript II First-Strand cDNA Synthesis Kit (Takara, Cat^#^ RR036A) and GeneAmp PCR system 9700 (Applied Biosystems, USA). The cDNA was then amplified by using specific primers (Supplemental material, Table S1) for detecting H1N1 in LightCycle 480 SYBR Green I Master (Roche, USA), and rhinovirus RNA was amplified with specific primers (Table S1) (25) by AgPath-ID One-Step RT-PCR (Applied Biosystems, Cat^#^ 4387424) (26). For quantitation, 10-fold serial dilutions of the standard plasmid equivalent to 10^1^ to 10^6^ copies per reaction were prepared to generate the calibration curve. The qPCR experiments were performed using the LightCycler 96 system (Roche, USA).

### Cytotoxicity assay

Cytotoxicity of peptides was determined by detecting 50% cytotoxic concentration (CC50) using a tetrazolium-based colorimetric MTT assay (27). Cells were seeded in a 96-well cell culture plate at an initial density of 4 × 10^4^ cells per well in MEM, supplemented with 10% FBS and incubated overnight. Cell culture media were removed, and then MEM supplemented with various concentrations of peptides and 1% FBS was added to each well. After 24 h incubation at 37°C, the MTT solution (5 mg mL^−1^, 10 µL per well) was added to each well for incubation at 37°C for 4 h. Then, 100 µL of 10% SDS in 0.01M HCl was added to each well. After further incubation at room temperature with shaking overnight, the plates were read at OD_570_ using the VictorTM X3 Multilabel Reader (PerkinElmer, USA). Cell culture wells without peptides were used as the experiment control, and the medium was a blank control.

### Viral attachment assay

Viruses (H1N1 or rhinovirus A1) were treated with DMEM, 4H30 (25 µg mL^−1^), or an influenza neutralization antibody at room temperature for 30 min and then added to cells for attachment at 4°C for 1 h. After washing the unbound virus, the attached virus on cells was measured by RT-qPCR. At least two experiments with biological duplication samples were included for each treatment.

### Viral entry assay

To identify the effect of 4H30 on viral entry, the H1N1 virus or rhinovirus A1 was prelabeled with green Dio dye (Invitrogen, Cat#3898) according to the manufacturer’s instructions. The labeled virus was purified by MicroSpin G-50 Columns (Cytiva, Cat^#^ 27533001). The Dio-labeled virus was treated with DMEM, 4H30 (25 µg mL^−1^), or H30 (25 µg mL^−1^) for 45 min. The pretreated H1N1 virus or rhinovirus infected MDCK or RD cells at 37°C or rhinovirus at 33°C for 1 h. Infected cells were fixed by 4% formalin at 1 hpi. The cell membrane was stained by membrane dye Alexa 594 (red, Invitrogen, W11262), and cell nuclei were stained by DAPI (blue). Entry or no entry of the virus on the cell membrane was determined by a confocal microscope (Carl Zeiss LSM 800, Germany).

### Viral release assay

H1N1 virus or rhinovirus A1 (MOI = 0.5) was used to infect MDCK or RD cells at 37°C or 33°C. At 5 hpi or 14 hpi, DMEM or drugs (4H30, 25.0 µg mL^−1^) or (zanamivir, 5 µM) were added to viral culture supernatants. At 8 hpi or 18 hpi, supernatants and cell lysates were collected for measuring viral titers by RT-qPCR. Two or three independent experiments with biological duplication samples were included for each treatment.

### HA immunostaining

MDCK cells were infected with H1N1 virus (0.5 MOI) at 37°C. After 14 h infection, viral culture supernatants were removed, and fresh DMEM with or without 4H30 (12.5 µg mL^−1^) or zanamivir (5 µM) was added to infected cells. At 18 hpi, cells were fixed at room temperature for 1 h and then were blocked by 5% BSA for 1 h. Mouse-anti-HA (Sino, Cat^#^ 11055-MM08, 1: 2,000) and goat anti-mouse IgG Alexa-488 (Life Technologies, Cat^#^ A32723, 1:600) were incubated with cells at room temperature for 45 min to stain viral HA protein. Images were taken by using a confocal microscope (Carl Zeiss LSM 800, Germany).

### HA-mediated cell fusion

The 293T cells were co-transfected with pGFP and pH7N7-HA (20). At 24 h post-transfection, cells were treated with PBS or FBP (125 µg mL^−1^) or 4H30 (125 µg mL^−1^) for 1 h and then treated with pH 5.0 or pH 7.4 for 10 min. After removing the pH buffer, cells were cultured at 37°C for 4 h with complete media. Fusion pictures were taken at 4 h after pH treatment.

### ELISA binding assay

Peptides (1.0 µg per well) were dissolved in H2O. Influenza HA, NA (Sino, Cat^#^ 11058-V07B), or LDLR (Sino, Cat^#^ 10231-H081-50, 100 ng) dissolved in PBS was coated onto ELISA plates, incubated at 4°C overnight, and blocked with BSA at 4°C overnight. HA or VP1 was added to wells for binding and then determined by incubation with goat anti-His-HRP (Invitrogen, Cat^#^ R93125, 1: 2,000) at 37°C for 30 min. The reaction was developed by adding 100 µL of the TMB single solution (Life Technologies, Cat^#^ 002023) for 15 min at 37°C and stopped with 50 µL of 1 M H_2_SO_4_. Readings were obtained in an ELISA plate reader (Victor 1420 Multilabel Counter; PerkinElmer) at 450 nm.

### ChABC and Hase treatment assay

MDCK cells were treated by ChABC (Sigma, Cat^#^ C2905, 1 U mL^−1^), Hase I–III (Sigma, Cat^#^ H3917, 100 mU mL^−1^), and Hase II (Sigma, Cat^#^ H6515, 100 mU mL^−1^) at 37°C for 2 h in the buffer according to the manufacturer’s instruction. After washing cells with PBS, MEM or 4H30 (25 µg mL^−1^) was added to cells for 30 min incubation, and then cells were washed with PBS. H1N1 (MOI = 1.0) was added to cells for attachment at 4°C for 1 h. The unbound virus was washed with PBS. The concentration of the attached virus was measured by RT-qPCR.

To investigate the effect on viral release, MDCK cells were infected with H1N1 (MOI = 1.0) at 37°C and then treated with ChABC, Hase I-III, and Hase II at 4 hpi in the buffer according to the manufacturer’s instruction. After 2 h incubation and washing of cells with PBS, DMEM with or without 4H30 ( 25 µg mL^−1^) was added to cells for virus culture. At 10 hpi, viral titers in cell supernatants were measured by RT-qPCR.

### Anti-H1N1 assay in mice

BALB/c mice (female, 8–10 weeks) were kept in a biosafety level 2 laboratory (housing temperature between 22 and 25°C with dark/light cycle) and given access to standard pellet feed and water *ad libitum*. All experimental protocols followed the standard operating procedures of the approved biosafety level 2 animal facilities. Animal ethical regulations were approved by the Committee on the Use of Live Animals in Teaching and Research of the University of Hong Kong (59-8721). To evaluate the post-exposure antiviral activity, mice were intranasally inoculated with H1N1 virus (A/Hong Kong/415742Md/2009). At 8 hpi, PBS or 4H30 (0.5 mg kg^−1^) was intranasally given to mice. Two more doses were given to mice on the following day. Viral loads in the lungs were measured by plaque assay. Body weight changes were measured every day until day 14 post-infection. Ten mice in each group were included to generate the final survival data.

### Anti-rhinovirus assay in cardiomyocytes

Cardiomyocytes are generated from human-induced pluripotent stem cells (iPSCs) as we had previously published (22, 23). Initially, IMR90 iPSCs are maintained in 12-well plates pre-coated with Matrigel (Thermo Fisher Scientific, USA) using StemFlex Medium (Thermo Fisher Scientific, USA). Subsequently, differentiation into cardiomyocytes is induced with the STEMdiff Ventricular Cardiomyocyte Differentiation Kit (Stem Cell Technologies, USA). This involves the sequential addition of Supplement A from days 1 to 2, Supplement B from days 3 to 4, and Supplement C from days 5 to 8 to cardiomyocyte differentiation basal medium. From day 9 onward, cardiomyocytes are sustained in cardiomyocyte maintenance basal medium with supplement (Stem Cell Technologies, USA). Lactate purification of cardiomyocytes is performed on days 11 to 12. Standard immunofluorescence staining with α-actinin and electrophysiology analysis by the patch clamping technique was performed to confirm their cardiomyocyte phenotype. To infect the cells, Rhinovirus A1 (0.025 TCID_50_) was treated with or without peptide in the media for infection. After 1 hour of infection, the non-infected virus was removed, and fresh media with or without peptides were added to cells for viral culture. Supernatant viruses were collected at 1 hpi, 24 hpi, and 48 hpi to measure viral loads by RT-qPCR.

### Anti-rhinovirus assay in nasal organoids

Organoid culture was established according to previous studies (28, 29). When the cells differentiated into mature 2D airway organoids within 12–14 days, the 2D organoids were ready for antiviral experiments. Rhinovirus (5000 TCID_50_) was mixed with peptide (25 µg mL^−1^) or without peptides in culture media for organoid infection. After 2 h infection, we removed the infectious media and added fresh media with or without peptides. The supernatants were collected at 2 hpi, 24 hpi, 48 hpi, and 72 hpi to measure the viral loads by RT-qPCR.

### Statistical analysis

The statistical significance of the results was calculated by the two-tailed Student *t*-test. Results were considered significant at *P* < 0.05.

## RESULTS

### Human defensin peptide 4H30 inhibits influenza virus entry

**Fig 1:**
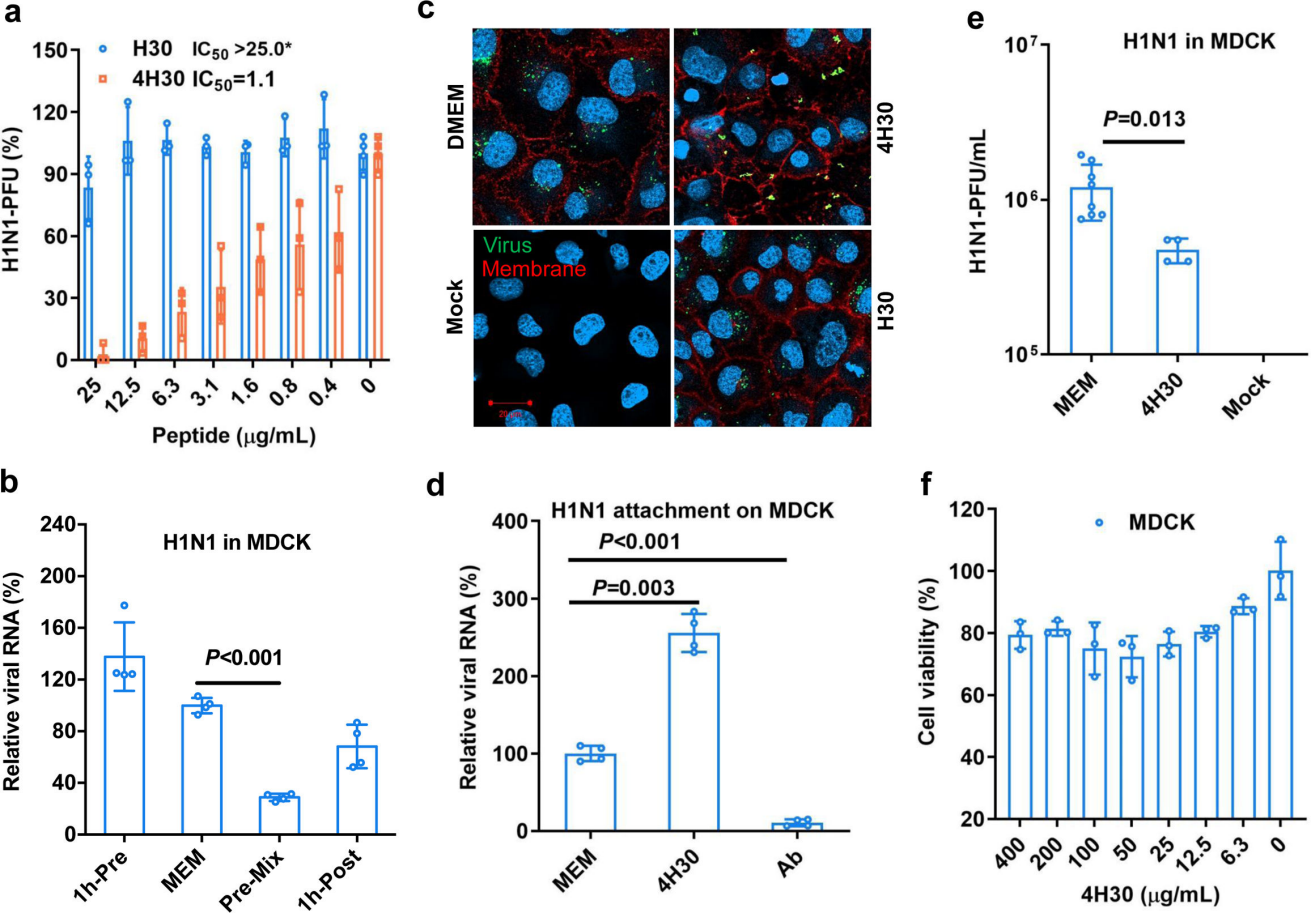
4H30 cross-linked H1N1 and blocked viral entry. (a) The antiviral activity of H30 and 4H30 against H1N1 virus in MDCK cells. IC_50_ of 4H30 against H1N1 is 1.1 µg mL^−1^. (b) Compared to the MEM-treated virus, 4H30 (25 µg mL^−1^) significantly inhibited H1N1 virus when the virus was treated before viral infection at 37°C, but 4H30 did not inhibit virus when cells were treated with 4H30 at 1 h before viral infection (1h-Pre) and cells treated with 4H30 at 1 h after viral infection (1h-Post). Viral RNA copies in cell lysates were measured at 6 hours post-infection (hpi). (c) 4H30 (25 µg mL^−1^) cross-linked virus and blocked viral entry into cells. Nuclei were stained by DAPI (blue). Cross-linked viruses (green) stuck on the cell membrane (red) are shown in yellow. Scale bar = 20 µm. (d) 4H30 (25 µg mL^−1^) increased viral binding to MDCK cells at 4°C. H1N1 neutralizing antibody (Ab) was used as a control. (e) 4H30 showed significant antiviral activity by targeting the virus. Virus was treated with 4H30 (50 µg mL^−1^) and then was diluted 1000-fold to decrease the concentration of 4H30 to 0.05 µg mL^−1^ for plaque assay. (f) The cytotoxicity of 4H30 in MDCK was evaluated by the MTT assay. * indicates *P* < 0.05. Data are presented as mean ± SD of indicated biological samples.

We first demonstrated that 4-branched H30 (4H30), but not the linear H30, could significantly inhibit group 1 influenza H1N1 virus infection in MDCK cells using plaque reduction assay (Fig. 1a). H30 could show antiviral activity in the low salt condition of 20% phosphate-buffered saline (20% PBS) compared to H30 in the high salt condition of PBS (Fig. S1), consistent with the typical salt sensitivity observed in defensin peptides (15, 30). We further demonstrated that 4H30 significantly inhibited group 2 influenza H3N2 virus infection (Fig. S2). To investigate the antiviral mechanism, we found that 4H30 inhibited viral replication when the virus was treated with 4H30 before viral infection (Fig. 1b). However, it did not show significant antiviral activity when cells were treated before and after viral infection. Through fluorescence microscopic images, we illustrated that 4H30 prevented viral entry into cells, and the aggregated viral particles with large sizes were stuck on the cell membrane without an entry (Fig. 1c). We further demonstrated that 4H30 increased the viral attachment to cells (Fig. 1d), which was consistent with the images of H1N1 virus clustering on the cell membrane (Fig. 1c). This finding aligns with our earlier discovery that 4H30 binds to cell surface GAGs (30) and subsequently cross-links virus with GAGs through noncovalent binding, affecting viral attachment. Moreover, when the H1N1 virus was treated with 4H30 (50 µg mL^−1^) and then diluted 1,000-fold to reduce the 4H30 concentration to 0.05 µg mL^−1^ (below the IC_50_ of 4H30 at 1.1 µg mL^−1^), 4H30 displayed significant antiviral activity against the virus (Fig. 1e). This indicated that 4H30 effectively targeted the H1N1 virus to exert its antiviral effect. The cytotoxicity assay demonstrated that 4H30 did not show noticeable toxicity in MDCK cells (Fig. 1f). These results indicated that 4H30 mainly inhibited viral entry and infection by targeting the virus.

### 4H30 blocks influenza virus release

**Fig 2:**
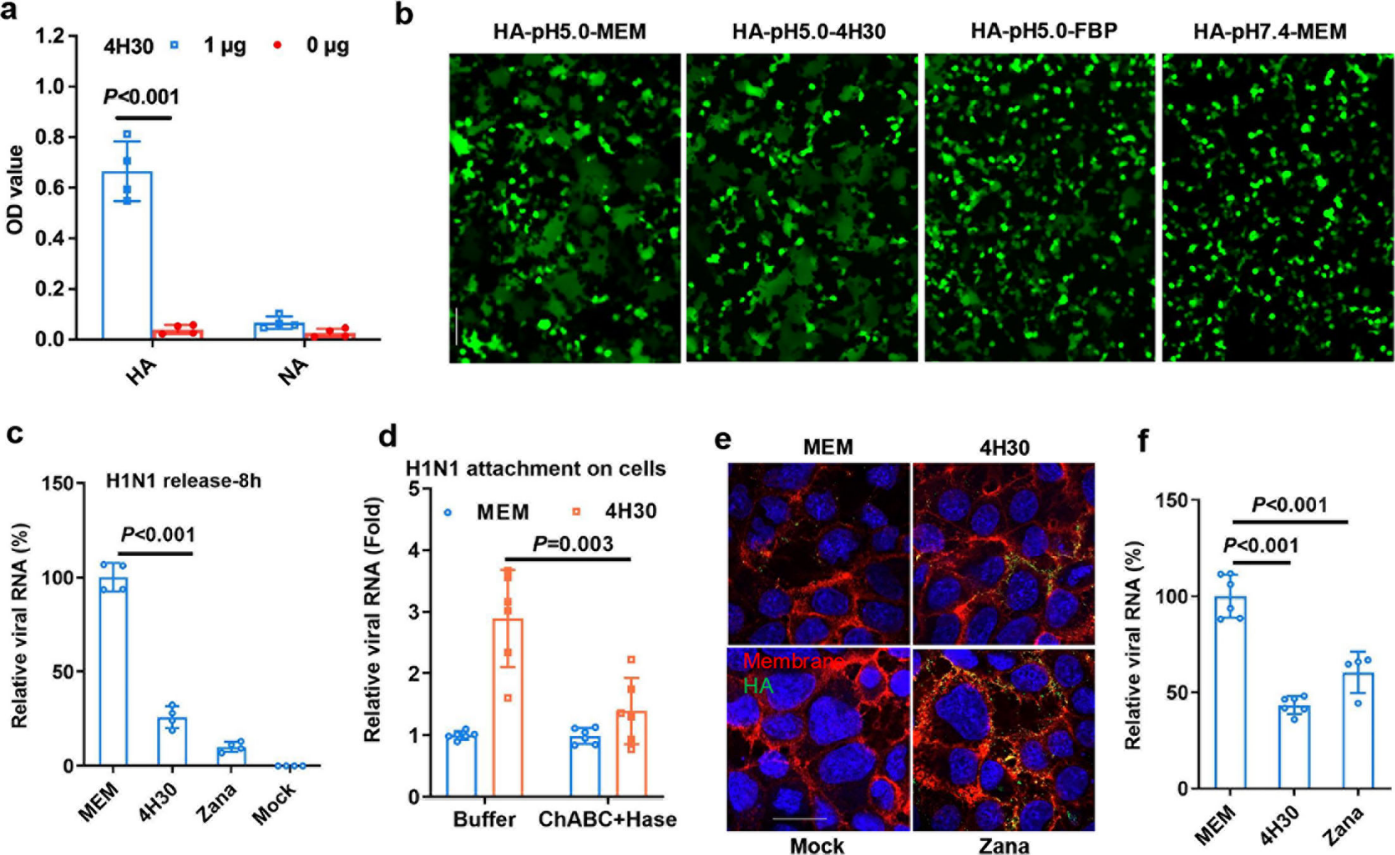
4H30 bound to H1N1 virus and cross-linked the virus with cellular GAGs to inhibit viral release. (a) 4H30 more efficiently bound to HA protein than NA. 4H30 (1 µg and 0 µg) was coated to an ELISA plate, and HA and NA (100 ng) binding to 4H30 was detected by anti-His antibody. (b) 4H30 (125.0 µg mL^−1^) did not inhibit HA-mediated cell-cell fusion in 293T cells. Scale bar = 200 µm. (c) 4H30 inhibited H1N1 release from MDCK cells. H1N1-infected cells were treated with MEM, 4H30 (25.0 µg mL^−1^), or zanamivir (5.0 µM) at 1 hour post-infection (hpi). The viral RNAs in supernatants were measured by RT-qPCR at 8 hpi. (d) Removing cell surface GAGs reduced the attachment of viruses treated with 4H30. MDCK cells treated with buffer (Buffer), and MDCK cells treated with Hase and ChABC to remove cellular GAGs were treated with MEM or 4H30 (25 µg mL^−1^) for H1N1 virus attachment at 4°C. Viral RNAs from cell lysates were measured by RT-qPCR. (e) 4H30 inhibiting viral release was confirmed by anti-HA staining. H1N1-infected cells were treated with MEM, 4H30, or zanamivir at 14 hpi and fixed at 18 hpi for anti-HA staining. HA (green) overlapped with the cell membrane (red) is shown in yellow. Scale bar = 20 µm. (f) 4H30 inhibited viral release at 18 hpi. This experimental procedure was the same as described in (e). The supernatant virus at 18 hpi was measured by RT-qPCR. Data are presented as mean ± SD of indicated biological samples.

To further investigate the antiviral mechanism, we demonstrated that 4H30 bound to HA protein, but not effectively bound to NA (Fig. 2a). However, 4H30 did not effectively block HA-mediated cell-cell fusion induced by low pH (Fig. 2b) compared to fusion inhibition peptide FBP (31). We further demonstrated that 4H30 and zanamivir significantly inhibited H1N1 viral release from cells compared to the untreated virus control (Fig. 2c), in which the supernatant virus was measured at 8 hours post-infection (hpi). It was consistent with the activity of 4H30 cross-linking virus with cell surface GAGs (30) to affect viral attachment and release. To further confirm whether 4H30 could cross-link H1N1 virus and cell surface GAGs, we demonstrated that the increase in H1N1 attachment to cells induced by 4H30 was significantly reduced when cells were treated with enzymes of Hase and ChABC to remove cellular GAGs (Fig. 2d). Additionally, the inhibition of viral release by 4H30 was further confirmed by anti-HA staining at 18 h post-infection (Fig. 2e), where more viral HA was detected on the cell membrane compared to the untreated virus, similar to the effect of zanamivir in blocking viral release. This result was further confirmed by RT-qPCR analysis at 18 hpi (Fig. 2f). Moreover, the viral neuraminidase assay indicated that 4H30 did not affect neuraminidase activity compared to zanamivir (Fig. S3), while the removal of GAGs reduced the activity of 4H30 in inhibiting viral release (Fig. S4). In summary, these results indicated that 4H30 blocked influenza virus entry by aggregating viral particles and inhibited viral release by cross-linking virus with cell surface GAGs.

### 4H30 protects mice from A(H1N1)pdm09 virus challenge

**Fig 3:**
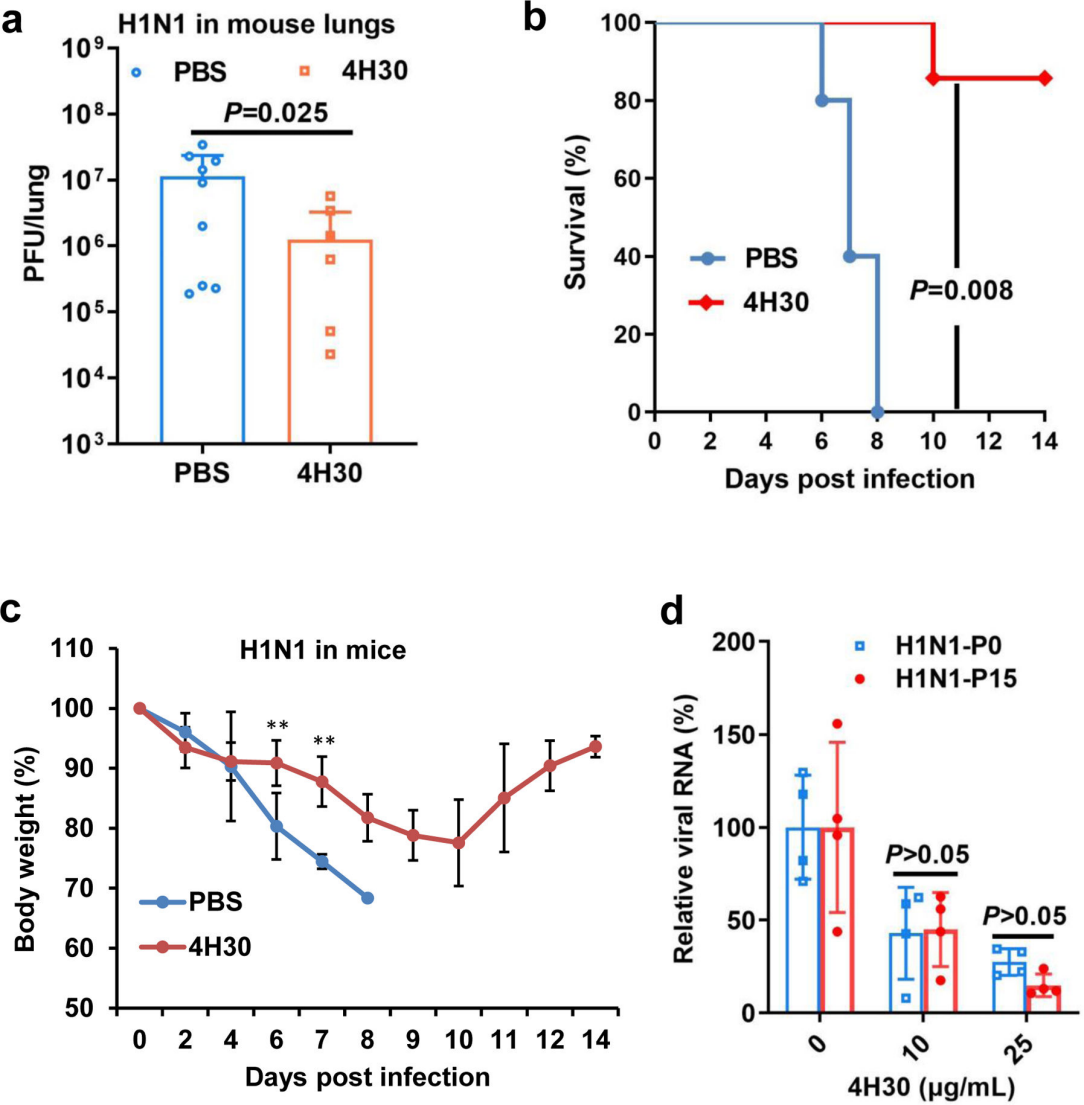
Protection of 4H30 on H1N1-infected mice and drug-resistance testing of 4H30 against H1N1 virus. (a) 4H30 significantly inhibited H1N1 replication in mouse lungs at 48 hpi (*n* = 6–9). (b) 4H30 increased the survival of mice infected with a lethal dose of H1N1 infection (*n* = 6). (c) The body weight changes of H1N1-infected mice. H1N1 virus was intranasally inoculated to mice, and then PBS or 4H30 (0.5 mg kg^−1^) was intranasally inoculated to mice at 6 hpi. On the following day, two more doses were given to mice. (d) 4H30 did not induce a drug-resistant virus after 15 passages. H1N1 virus was passaged in the presence of 4H30 (2.5, 5.0, and 10.0 μg/mL^−1^ for every five passages). The 15-passage virus (P15) was not resistant to 4H30 compared to the antiviral activity of 4H30 against the parental virus (P0). ** indicates *P* < 0.01. Data are presented as mean ± SD of indicated biological samples.

To assess the antiviral activity of 4H30 *in vivo*, mice were intranasally inoculated with 4LD_50_ of A(H1N1)pdm09 virus. At 6 hpi, mice were intranasally inoculated with 4H30 (0.5 mg/kg), and two more doses were administered to mice the following day. We first tested the viral loads in the lungs on day 2 post-infection (Fig. 3a), revealing that 4H30 significantly inhibited H1N1 viral replication in mouse lungs compared to PBS-treated mice. Subsequently, we examined the survival of mice infected with A(H1N1)pdm09 virus (Fig. 3b), demonstrating that 4H30 significantly protected mice (5/6) from the lethal challenge of H1N1 virus. Analysis of body weight data indicated that 4H30 significantly reduced body weight loss compared to PBS-treated mice after day 4 post-infection (Fig. 3c). The toxicity assay *in vivo* (30) and *in vitro* (Fig. 1f) indicated that 4H30 significantly inhibited A(H1N1)pdm09 virus replication without causing apparent toxicity both *in vitro* and in mice. Furthermore, we investigated the resistance barrier of 4H30 against the H1N1 virus by passaging the virus in the presence of 4H30. After 15 passages (P15), no drug-resistant H1N1 virus was detected (Fig. 3d), in which 4H30 showed consistent activity against both H1N1-P0 and H1N1-P15. In contrast, zanamivir-resistant H1N1 virus could be generated after four passages in the presence of zanamivir (24, 32). These results indicated that 4H30 significantly inhibited A(H1N1)pdm09 viral replication in mice, increased the survival of H1N1-infected mice, and exhibited a high barrier to viral resistance.

### 4H30 inhibits rhinovirus entry in RD cells

**Fig 4:**
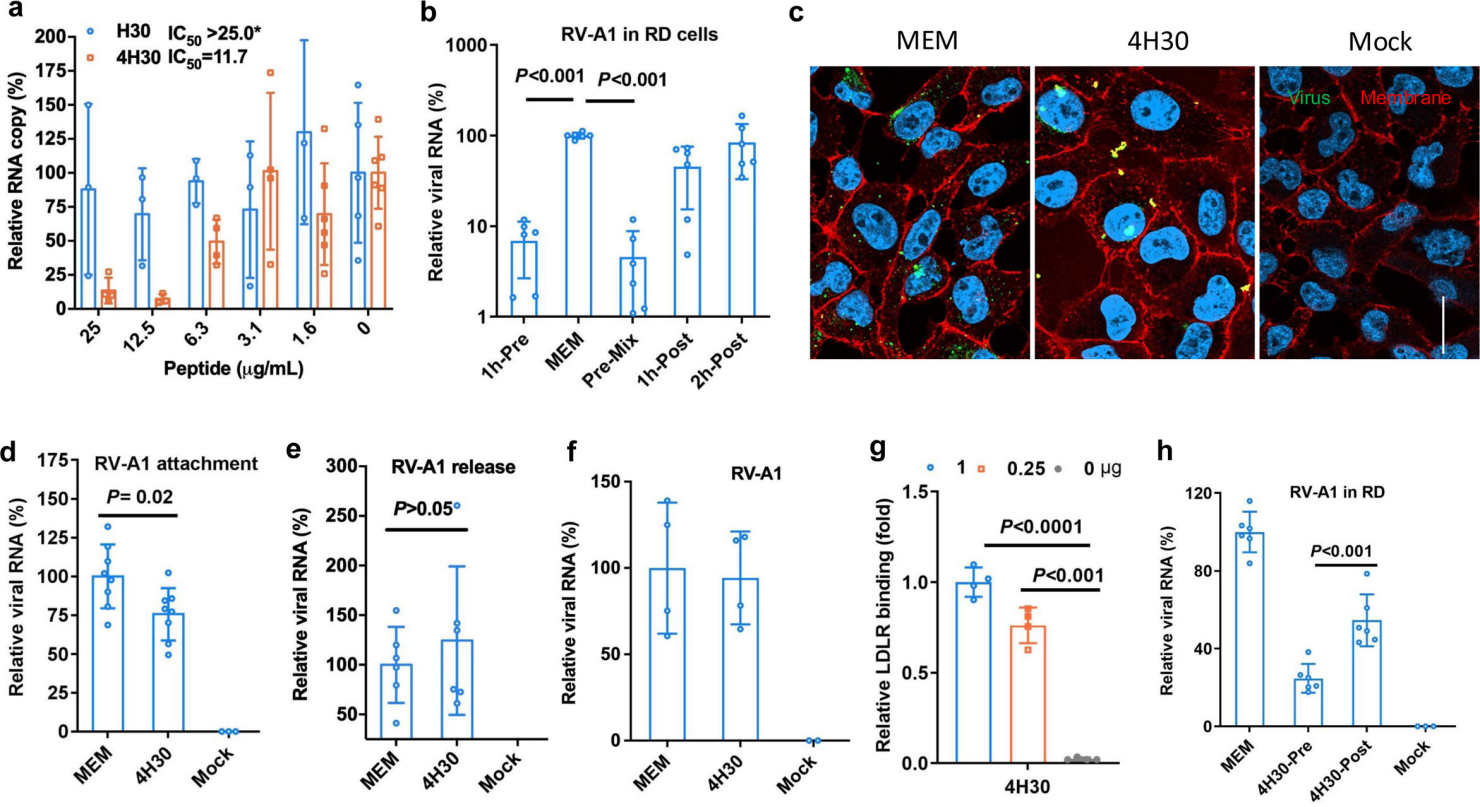
4H30 inhibited rhinovirus entry. (a) 4H30 (25.0 µg mL^−1^) significantly inhibited rhinovirus replication in RD cells. Virus was treated with the indicated concentrations of peptides, and viral RNA in the supernatants was measured at 30 hpi. (b) 4H30 significantly inhibited rhinovirus A1 (RV-A1) replication when cells were treated with 4H30 at 33°C for 1 h before viral infection (1h-Pre), and the virus was treated before infecting cells (Pre-mix). When infected cells were treated with 4H30 at 1 hpi (1h-Post) or 2 hpi (2h-Post), it did not inhibit viral infection. Viral RNAs in supernatants were measured at 16 hpi. (c) 4H30 (25.0 µg mL^−1^) cross-linked rhinovirus and blocked viral entry into RD cells. Nuclei were stained with DAPI (blue). Scale bar = 20 µm. (d) 4H30 reduced viral attachment on RD cells at 4°C when virus was treated with 4H30. (e) 4H30 (25.0 µg mL^−1^) did not inhibit viral release from RD cells. RD cells were infected with RV-A1 and were treated with 4H30 at 20 hpi. The supernatant viral RNAs were measured at 30 hpi. (f) 4H30 did not inhibit rhinovirus A1 (RV-A1) by targeting the virus. Rhinovirus A1 was treated with 4H30 (100 µg mL^−1^) in PBS for 1 h. After 1 hour of treatment, the treated virus was diluted in MEM with 200-fold to let the concentration of 4H30 decrease to 0.5 µg mL^−1^. The diluted virus was used to infect RD cells at 33°C. Viral RNA copies in cell lysates were measured at 16 hpi. No viral infection was the mock control. (g) 4H30 (1.0 µg, 0.25 µg, and 0 µg) bound to LDLR in the ELISA assay. (h) 4H30 showed significant antiviral activity before the virus was bound to RD cells. The virus was added to cells in the presence of 4H30 (25 µg mL^−1^) (4H30-Pre) or without 4H30 to infect cells at 4°C. After 1 h attachment, the virus was removed, and fresh MEM or MEM with 4H30 (4H30-Post) was added to cells for viral culture at 33°C. Viral RNA copies in cell lysates were measured at 16 hpi. * indicates *P* < 0.05. Data are presented as mean ± SD of indicated biological samples.

To investigate the broad antiviral activity, we demonstrated that 4H30, but not H30, significantly inhibited rhinovirus infection in RD cells (Fig. 4a). Notably, H30 could show more effective antiviral activity in low salt conditions (Fig. S5). To investigate the antiviral mechanism, we first demonstrated that 4H30 significantly inhibited viral replication when cells or viruses were treated with 4H30 before viral infection (Fig. 4b), but 4H30 showed reduced antiviral activity when cells were treated after viral infection (1h-Post and 2h-Post). Subsequently, we demonstrated that 4H30 blocked rhinovirus entry on the cell membrane (Fig. 4C), consistent with its antiviral activity of inhibiting viral replication when cells were treated before viral infection. 4H30 reduced rhinovirus attachment to RD cells (Fig. 4d), but did not inhibit viral release (Fig. 4e). Moreover, when rhinovirus was treated with 4H30 (50 µg mL^−1^) and then the treated virus was 100-fold diluted to reduce the 4H30 concentration to 0.5 µg mL^−1^ for infection, 4H30 did not show antiviral activity against rhinovirus (Fig. 4f). This indicated that 4H30 did not rely on targeting rhinovirus to inhibit viral infection, unlike its activity against the H1N1 virus (Fig. 1e), where it increased viral attachment (Fig. 1d) and inhibited viral release (Fig. 2c). We further demonstrated that 4H30 bound to the LDLR receptor of rhinovirus (Fig. 4g), explaining its significant antiviral activity when cells were treated with 4H30 before viral infection (Fig. 4b) and its ability to block rhinovirus entry (Fig. 4c). Additionally, we found that 4H30 showed significant antiviral activity when treating virus during viral infection (without the need for pretreating virus), and its antiviral activity diminished when rhinovirus was attached to cells at 4°C and was treated with 4H30 at 1 hpi (Fig. 4h). This result indicated that 4H30 more efficiently inhibited rhinovirus replication before virus binding to LDLR, even when maintained in the culture media, indicating that 4H30 targets LDLR to exert its antiviral activity. To investigate the antiviral activity of 4H30 relying on LDLR, we demonstrated that 4H30 inhibited rhinovirus A1 in H1-Hela cells but did not show antiviral activity against rhinovirus A16, which uses hICAM-1 as the receptor (Fig. S6). The cytotoxicity assay showed that the TC_50_ of 4H30 in RD cells was higher than 400 µg mL^−1^ (Fig. S7). These results indicated that 4H30 significantly inhibited the cell entry of minor group rhinovirus by targeting the LDLR receptor of host cells.

### 4H30 inhibits rhinovirus replication in nasal organoids and cardiomyocytes

**Fig 5:**
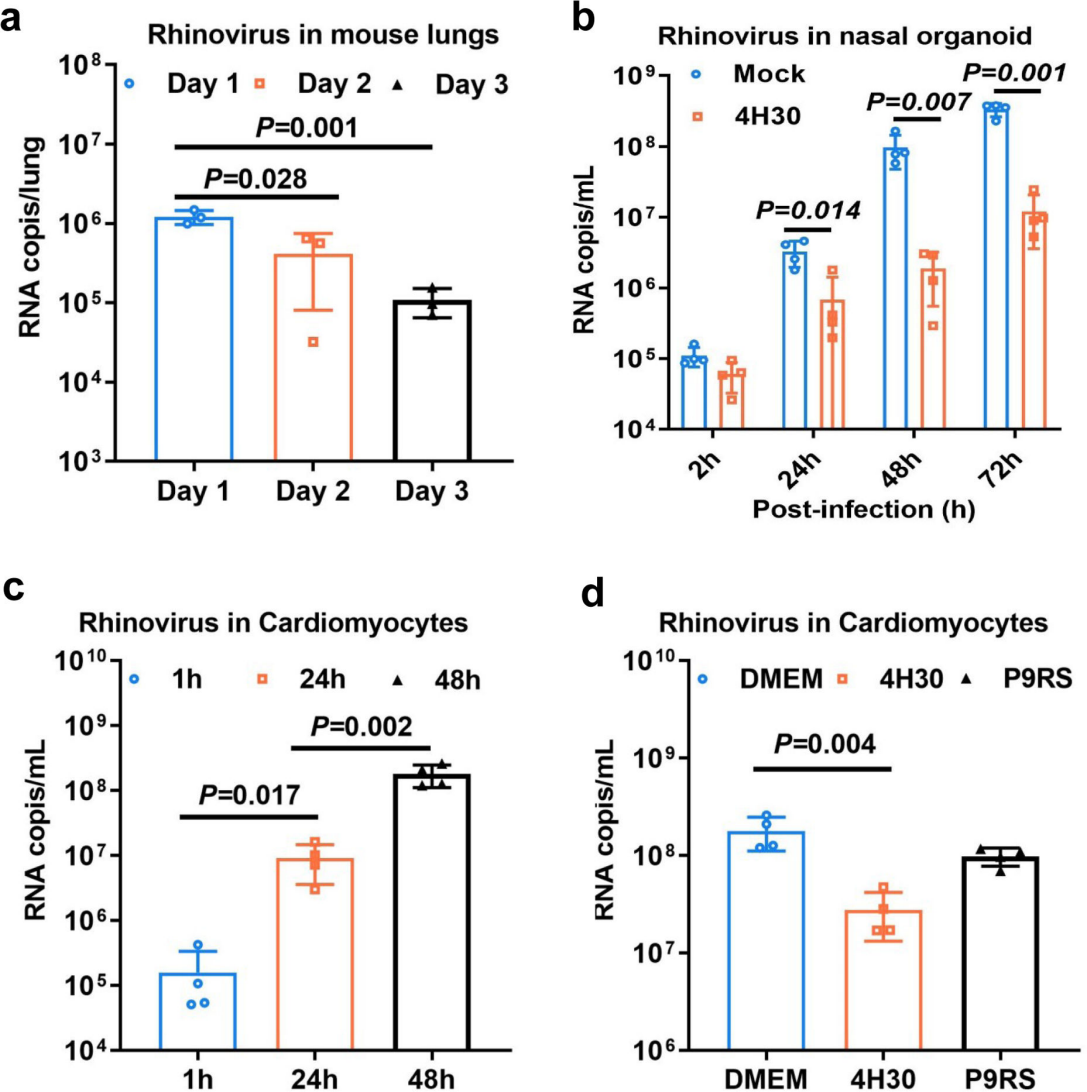
4H30 inhibited rhinovirus replication in nasal organoids and cardiomyocytes. (a) Rhinovirus A1 could not replicate efficiently in mice. Rhinovirus (1 × 10^7^ TCID_50_) was intranasally inoculated to mice. Viral RNAs were measured on day 1, day 2, and day 3 post-infection. (b) Rhinovirus A1 (0.01 MOI) could efficiently replicate in nasal organoids, and 4H30 (25 µg mL^−1^) could significantly inhibit viral replication. (c) Rhinovirus A1 (0.01 MOI) could effectively replicate in cardiomyocytes. (d) 4H30 (25 µg mL^−1^) could significantly inhibit rhinovirus replication in cardiomyocytes at 48 phi. Viral RNAs in the cell supernatants were measured by RT-qPCR at the indicated time points. Data are presented as mean ± SD of indicated biological samples.

Because the existing animal models cannot support the efficient proliferation of rhinovirus for antiviral assay (33) (Fig. 5a), we did not evaluate the antiviral efficacy of 4H30 in mice. To comprehensively investigate the antiviral efficacy of 4H30 in different models, we used the nasal organoids (29) to demonstrate efficient rhinovirus replication, with an around 1,000-fold increase in viral loads in the supernatants from 2 hpi to 72 hpi (Fig. 5b). Subsequently, we showed that 4H30 significantly inhibited rhinovirus replication in nasal organoids by reducing viral loads in supernatants at 24 hpi, 48 hpi, and 72 hpi (Fig. 5b), further confirming the antiviral activity of 4H30 in the human upper respiratory tract. Considering the reported association between rhinovirus and dilated cardiomyopathy (34, 35), we employed human-induced pluripotent stem cells derived cardiomyocytes (hIPSC-CMs) as a cardiomyocyte model (Fig. S8) to investigate rhinovirus replication. Our results revealed that rhinovirus effectively replicated in hIPSC-CMs, with viral increases exceeding 1,000 folds from 1 hpi to 48 hpi (Fig. 5c), suggesting the potential for rhinovirus infection in heart-sourced cells. Furthermore, we demonstrated that 4H30 significantly inhibited viral replication in hIPSC-CMs (Fig. 5d) compared to DMEM- and P9RS-treated controls. In addition to cancer cell lines and stem cell-derived cardiomyocytes, nasal organoids as an upper respiratory tract model of human source could be an effective cell model to evaluate the antiviral efficacy of drugs. These results indicated that 4H30 significantly inhibited rhinovirus replication in RD cells, hIPSC-CMs, and nasal organoids.

## DISCUSSION

In this study, we demonstrated that the branched 4H30 exhibited broad-spectrum inhibitory activity against influenza virus and rhinovirus. Combined with our previous research on 4H30 against SARS-CoV-2 (30), these findings indicated that the branched peptide could be a valuable method to increase the antiviral activity of defensin peptides, which generally show limited antiviral efficacy in high salt conditions. Therefore, developing methods to improve the antiviral activity of defensin peptides could have widespread utility in antiviral drug design. Supported by this and our previous studies related to the efficacy of branched peptides against SARS-CoV-2 (30, 36) and the inherent advantages of branched peptides such as high stability *in vivo* and resistance to proteases (19, 37), we propose that branched peptides could be a promising method to increase the antiviral activity of defensin peptides and confer defensin peptides with potent antiviral activity in a salt-insensitive manner.

Influenza, rhinovirus, and SARS-CoV-2 cause millions of infections yearly. The current antiviral drugs have shown limited effectiveness against these viruses, leading to significant mortality rates associated with high pathogenic avian influenza viruses (1), as well as the highly transmissible influenza and SARS-CoV-2 infections (7, 11, 38), along with the emergence of drug-resistant viruses (8, 39). Discovering broad-spectrum antivirals is critical to combat viral mutant challenges and new emerging viruses. However, the quest for broad-spectrum antivirals presents challenges, including the risk of off-target side effects and the limited efficiency *in vivo*, such as bafilomycin A1, chloroquine, and camostat (31, 36, 40).

In this and our previous studies (30), we have demonstrated that the branched human defensin peptide 4H30 exhibits significantly greater antiviral activity than linear H30 *in vitro* and *in vivo*. The potent antiviral effects observed *in vitro* and in mice may be attributed to 4H30’s ability to cross-link the virus (targeting HA protein). 4H30 bound to cellular GAGs (41) and interacted with viral proteins (30). These dual-functional properties allow 4H30 to effectively block viral entry and suppress the release of viruses by cross-linking them with cell surface GAGs. Although 4H30 could not show anti-rhinovirus activity by targeting the virus, 4H30 significantly inhibited minor group rhinovirus by affecting LDLR to block viral entry. However, 4H30 was not effective against rhinovirus A16 from the major group, indicating that 4H30 could not interfere with the hICAM-1 receptor to inhibit this particular strain of rhinovirus. Given that both influenza virus and rhinovirus enter cells through the endocytic pathway and that endosomal pH inhibitors can impede viral infection, 4H30’ ability to inhibit pH decrease in endosomes (30) could interfere with viral infection by affecting pH decrease when the viruses enter cells via endosomes.

In conclusion, these findings highlight the potential of targeting both host factors (including GAGs, cellular receptor LDLR, and endosomal acidification) and viral surface proteins (influenza HA and SARS-CoV-2 spike) as an effective strategy to enhance the antiviral activity and maintain the broad-spectrum antiviral activity of human defensin peptide 4H30. The limited effectiveness of current antivirals against influenza virus, rhinovirus, and SARS-CoV-2 underscores the urgent need to discover new antivirals with innovative mechanisms. Broad-spectrum antiviral peptides with less metabolic toxicity and potent antiviral efficacy could be a promising and alternative addition to the current antiviral arsenal.

## Data Availability

The data supporting the findings of this study are available in the paper and its supplemental material.

## SUPPLEMENTAL MATERIAL

Supplemental material aac.00024-25-s0001.docx Fig. S1 to S8; Table S1.

ASM does not own the copyrights to Supplemental Material that may be linked to, or accessed through, an article. The authors have granted ASM a non-exclusive, world-wide license to publish the Supplemental Material files. Please contact the corresponding author directly for reuse.
