# Supplementary material for: A branched peptide targets virus and host to block influenza virus and rhinovirus entry
Source: Antimicrob Agents Chemother. 2025 Jun 25;69(8):e00024-25. doi: 10.1128/aac.00024-25 (PMC12326976; doi:10.1128/aac.00024-25)
Supplement: Supplemental material — Fig. S1 to S8; Table S1. [file aac.00024-25-s0001.docx]

**Supplementary information**

**A branched peptide targets virus and host to block influenza virus and rhinovirus entry**

Xinjie Meng, Chuyuan Zhang, Xiankun Wang, Jilong Shi, Zixian Song, Purui Ke, Yao Chen, Ruiqing Sun, Yee-Man Lau, Kwong-Man Ng, Chun-Ka Wong, Hung-Fat Tse, Linlei Chen, Kwok Hung Chan, Cyril Chik-Yan Yip, Jie Zhou, Youhua Xie, Shibo Jiang, Kelvin Kai-Wang To, Kwok-Yung Yuen, Hanjun Zhao*

**Table of contents**

Fig. S1. The antiviral activity of H30 against H1N1 virus in MDCK cells.

Fig. S2. The antiviral activity of 4H30 against H3N2 virus in MDCK cells.

Fig. S3. 4H30 did not inhibit the neuraminidase activity of H1N1 virus.

Fig. S4. The removal of GAGs reduced the inhibition of 4H30 on viral release.

Fig. S5. The antiviral activity of H30 against rhinovirus A1 in RD cells.

Fig. S6. 4H30 inhibited rhinovirus A1 but did not inhibit rhinovirus A16 in H1-Hela cells.

Fig. S7. The cytotoxicity of 4H30 in RD cells.

Fig. S8. Immunofluorescence staining of cardiomyocytes.

Table S1. Primers for qPCR


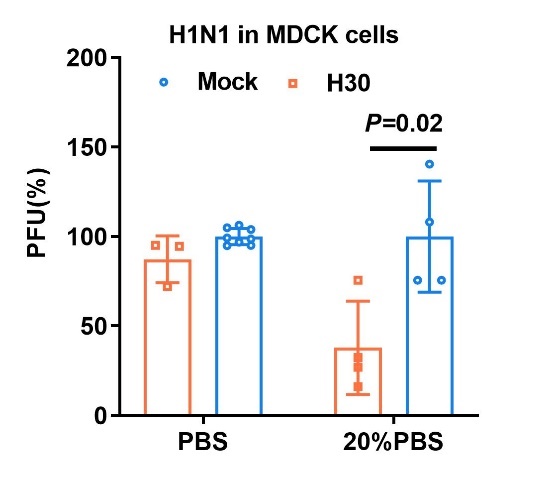


**Fig. S1. The antiviral activity of H30 against H1N1 virus in MDCK cells.** H1N1 virus was treated with H30 in PBS or 20% PBS for plaque assay. Viruses without treatment in PBS or in 20% PBS were the control. Data are presented as mean ± SD of indicated biological samples.


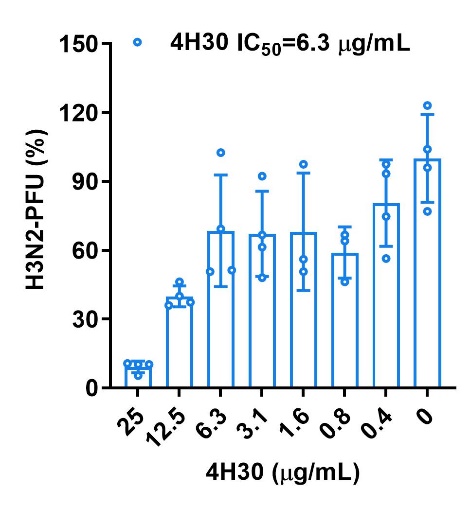


**Fig. S2. The antiviral activity of 4H30 against H3N2 virus in MDCK cells.** H3N2 virus was treated with the indicated concentrations of 4H30 to infect MDCK cells. The percentage (%) was the plaque number of virus treated with 4H30 normalized to virus without treatment (0).


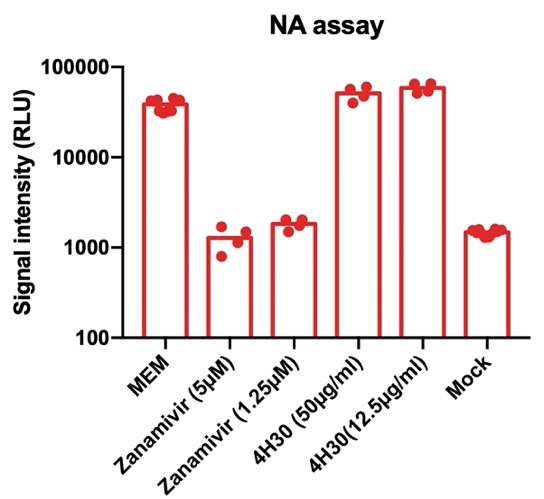


**Fig. S3. 4H30 did not inhibit the neuraminidase activity of H1N1 virus**. H1N1 virus was treated with MEM, zanamivir (5 μM and 1.25 μM), and 4H30 (50 μg mL^−1^and 12.5 μg mL^−1^) for 1 h. The neuraminidase activity was analysed using the Invitrogen NA-STAR INFLUENZA NIRD REAGENT (Cat^#^ 4374348). Data are presented as mean ± SD of indicated biological samples.


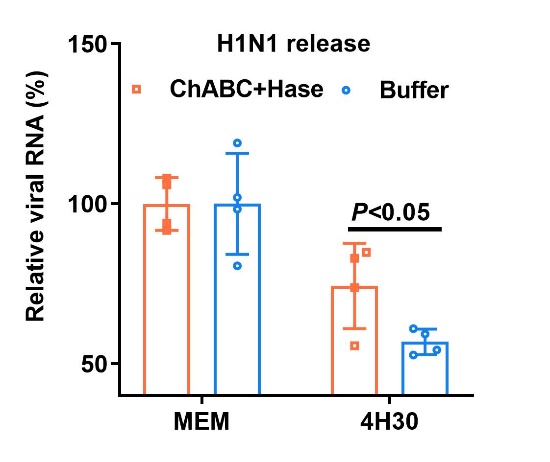


**Fig. S4. The removal of GAGs reduced the inhibition of 4H30 on viral release.** H1N1-infected MDCK cells were treated with buffer (Buffer) or treated with ChABC+Hase to remove cellular GAGs at 3 hpi. After 2 h treatment, cells were treated with MEM or 4H30 (25 μg mL^−1^) for viral culture. Vrial RNAs from supernatants at 10 hpi were measured by RT-qPCR. The inhibition of 4H30 on viral release was reduced when cells were treated with ChABC+Hase to remove GAGs.


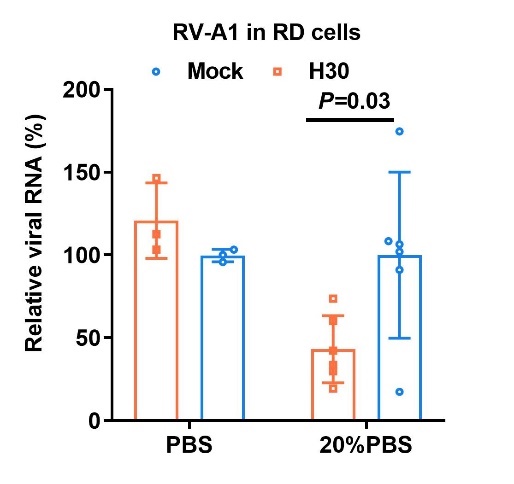


**Fig. S5. The antiviral activity of H30 against rhinovirus A1 in RD cells.** Rhinovirus A1 (RV-A1) was treated with H30 in PBS or 20% PBS to infect RD cells. After one hour of infection, infectious media were replaced with fresh MEM. Viral RNA copies in cells were measured at 16 hpi. Viruses without treatment in PBS or 20% PBS were the control. Data are presented as mean ± SD of indicated biological samples.

**a**

**b**


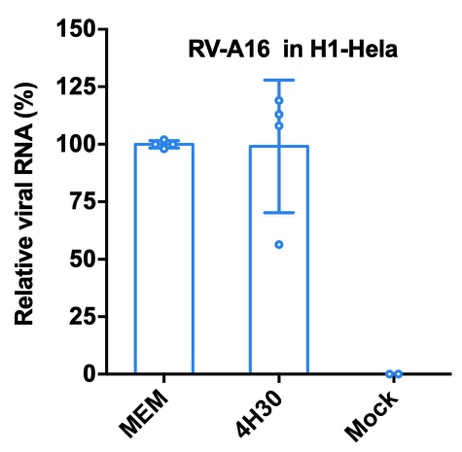

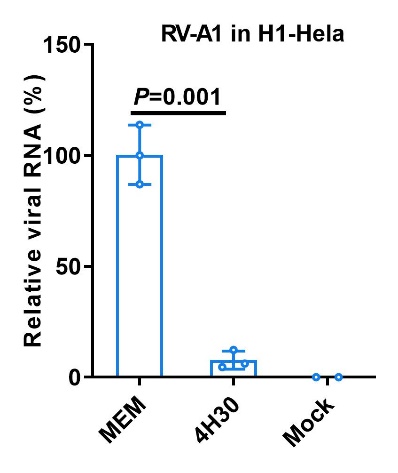


**Fig. S6. 4H30 inhibited rhinovirus A1 but did not inhibit rhinovirus A16 in H1-Hela cells.** H1-Hela cells are the standard cell model for efficient Rhinovirus A16 **(**RV-A16) replication. RV-A1 (**a**) or RV-A16 (**b**) was treated with 4H30 (25 μg mL^−1^) for one hour of infection and then the infectious media were replaced with MEM with 4H30 for viral culture. Viral RNA copies in cells were measured at 16 hpi. Virus in MEM was the control. No viral infection was the mock control. Data are presented as mean ± SD of indicated biological samples.


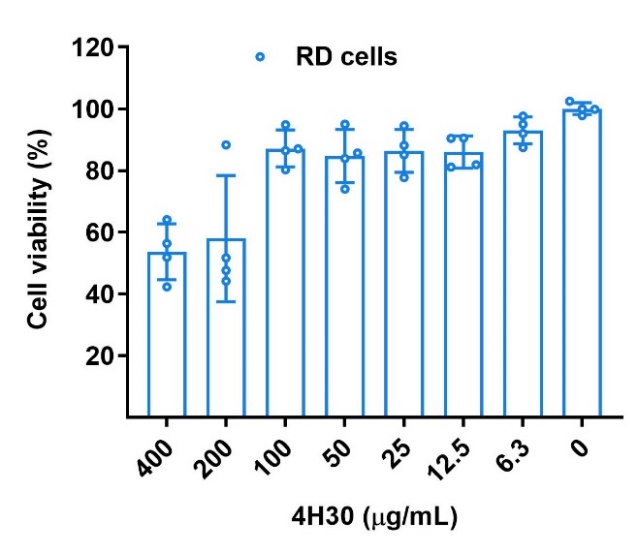


**Fig. S7. The cytotoxicity of 4H30 in RD cells.** RD cells were treated with 4H30 at the indicated concentrations. After 24h culture, cell viability was measured by MTT assay. Data are presented as mean ± SD of indicated biological samples.


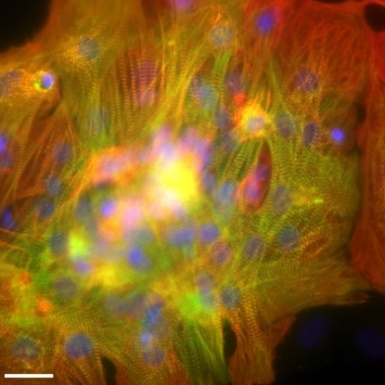

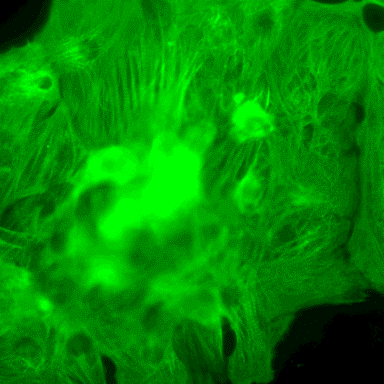

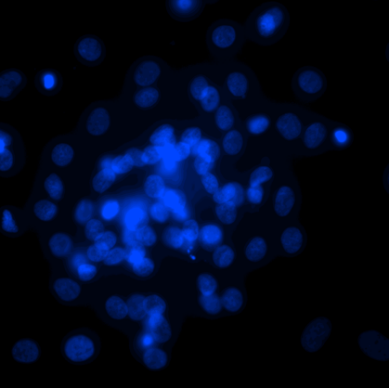

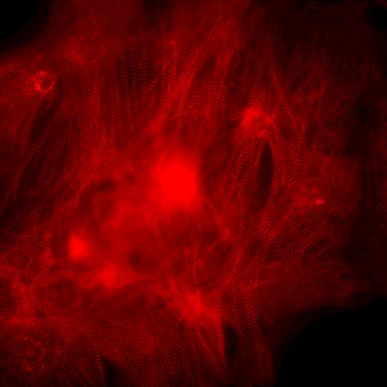


**Fig. S8.** **Immunofluorescence staining of cardiomyocytes.** After differentiation of induced pluripotent stem cells (iPSC) to cardiomyocytes with spontaneous beating, immunofluorescence staining with troponin T (green) and α-actinin (red) was performed to confirm cardiomyocytes phenotype. Nuclei were stained blue using 4',6-diamidino-2-phenylindole (DAPI). Scale bar represented 50 μm.

**Table S1. Primers for qPCR**

| Gene | Primer | Oligonucleotide sequence (5' to3') |
| --- | --- | --- |
| H1N1 | ^a^M-F | CTTCTAACCGAGGTCGAAACG |
|  | M-R | GGC ATTTTGGACAAAKCGTCT A |
| Rhinovirus | ^b^HRV-F1 | AGCCYGCGTGGCKGCC |
|  | HRV-F2 | AGCCYGCGTGGTGCCC |
|  | HRV-R | GAAACACGGACACCCAAAGTAGT |
|  | Probe | HEX-TCCGGCCCCTGAATGYGGCTAA-lABkFQ |

^a^: M gene primers for H1N1 virus qPCR. ^b^: human rhinovirus 5’UTR gene for rhinovirus RT-qPCR.
